# Estimating Tropical Forest Structure Using a Terrestrial Lidar

**DOI:** 10.1371/journal.pone.0154115

**Published:** 2016-04-28

**Authors:** Michael Palace, Franklin B Sullivan, Mark Ducey, Christina Herrick

**Affiliations:** 1 Earth System Research Center, Institute for the Study of Earth, Oceans, and Space, University of New Hampshire, Durham, New Hampshire, United States of America; 2 Department of Earth Sciences, University of New Hampshire, Durham, New Hampshire, United States of America; 3 Department of Natural Resources, University of New Hampshire, Durham, New Hampshire, United States of America; Chinese Academy of Forestry, CHINA

## Abstract

Forest structure comprises numerous quantifiable biometric components and characteristics, which include tree geometry and stand architecture. These structural components are important in the understanding of the past and future trajectories of these biomes. Tropical forests are often considered the most structurally complex and yet least understood of forested ecosystems. New technologies have provided novel avenues for quantifying biometric properties of forested ecosystems, one of which is LIght Detection And Ranging (lidar). This sensor can be deployed on satellite, aircraft, unmanned aerial vehicles, and terrestrial platforms. In this study we examined the efficacy of a terrestrial lidar scanner (TLS) system in a tropical forest to estimate forest structure. Our study was conducted in January 2012 at La Selva, Costa Rica at twenty locations in a predominantly undisturbed forest. At these locations we collected field measured biometric attributes using a variable plot design. We also collected TLS data from the center of each plot. Using this data we developed relative vegetation profiles (RVPs) and calculated a series of parameters including entropy, Fast Fourier Transform (FFT), number of layers and plant area index to develop statistical relationships with field data. We developed statistical models using a series of multiple linear regressions, all of which converged on significant relationships with the strongest relationship being for mean crown depth (*r*^*2*^ = 0.88, p < 0.001, RMSE = 1.04 m). Tree density was found to have the poorest significant relationship (*r*^*2*^ = 0.50, *p* < 0.01, *RMSE* = 153.28 n ha^-1^). We found a significant relationship between basal area and lidar metrics (*r*^*2*^ = 0.75, *p* < 0.001, *RMSE* = 3.76 number ha^-1^). Parameters selected in our models varied, thus indicating the potential relevance of multiple features in canopy profiles and geometry that are related to field-measured structure. Models for biomass estimation included structural canopy variables in addition to height metrics. Our work indicates that vegetation profiles from TLS data can provide useful information on forest structure.

## Introduction

Forest structure is a reflection of the principles of forest growth and disturbance, influenced by the spatial and temporal variability of resource availability, disturbance rates, and management [1–4]. The three dimensional architecture of a forest is a direct indication of ecosystem function, carbon and nutrient cycling, disturbance regimes, and the coupling between forests and regional and global climate [5]. Tropical forests have additional complexity in regard to species diversity and are thought to be among the most structurally complex of all forested ecosystems [6]. Tropical forest structure characterization is important in understanding ecological and earth system processes, knowledge of which proves vital in efforts to mitigate climate change through the reduction of greenhouse gases emissions. The ability to quantify forest structure beyond standing biomass is critical to efforts such as Reducing Emissions from Deforestation and Forest Degradation (REDD+), which depends on characterization of forest structure to provide insight into the previous carbon dynamics and the potential future storage capabilities of a forest [7–10]. Ground-based measurements allow for accurate mapping of vegetation structure, but only on very limited spatial and temporal scales, and with high costs and unknown biases [11].

The spatial variability of forest structure at the landscape scale is difficult to capture without remote sensing methods because proper evaluation of variability across the landscape would require extensive field campaigns, which can be cost prohibitive. To extract information related to changes in forest structure, measurements on the ground must be associated with those inferred from airborne and space-based remote sensing data [12–13]. Research and field sites that have both extensive field-based biometric data as well as remote sensing data are vital in estimating forest biometric properties. New technologies, such as LIght Detection And Ranging (lidar), offer the possibility of reducing inventory costs and increasing accuracy [14]. Lidar remote sensing has been used to estimate the horizontal and vertical heterogeneity in forest structure [15–19] and can be deployed from space, aircraft, and on the ground. Previous studies have demonstrated that airborne lidar-derived canopy vegetation profiles compare well with ground-based profiles [20–21]. Canopy vegetation profile metrics have been shown to be useful in predicting biomass and other structural forest properties [8, 17, 22–32]. A majority of effort has been to use discrete airborne lidar, yet ground-based lidar deployment may provide additional insight and is currently being deployed in many forested settings [33–37].

Ground-based imaging lidar systems often gather a detailed, three-dimensional digital model of forest stands and individual trees from an understory perspective [38]. These systems can be deployed quickly in multiple locations and gather information often faster than those collected by field crews and measure unique attributes often complementing field-based surveys [39–40]. In particular, ground-based lidar may be useful for reducing uncertainty associated with the generalized allometric equations used to convert standard tree measurements to biomass or carbon; error in allometrics is a key source of uncertainty in large-scale inventories, and does not decline with increased sampling intensity or number of plots [11, 41]. Terrestrial laser scanners were originally designed for precision surveying applications; applications to forest ecosystem measurement are just emerging [42]. Algorithms to estimate aboveground forest biomass, its components (foliage, stemwood, and branchwood), and their three-dimensional distribution are in their infancy but show great promise [43–45].

In this study, we focused on the utility of canopy profiles derived from TLS data for developing relationships to field data, similar to approaches that use airborne lidar scanners. Methodology for the calculation of RVPs is described in detail. We evaluated statistical relationships between TLS canopy profiles and associated metrics with field-measured biometric properties for twenty plots within La Selva Biological Station, Costa Rica. We used multivariate linear regression with stepwise variable selection to develop models of forest biometric properties from a suite of canopy profile metrics.

## Methods

### Study site and data sets

#### La Selva

We conducted our research at the La Selva Biological Station (10° 26’ N, 83° 59’ W), operated by the Organization for Tropical Studies and located in the Atlantic lowlands of Costa Rica [46]. We determined the location of twenty plots that were randomly selected to measure field-based biometric properties and collect ground-based terrestrial lidar scans. We selected our plot locations using a set of *a priori* constraints based on GIS data layers (trails existing studies, water bodies, vegetation type) provided by the La Selva (http://www.ots.ac.cr). Criteria for our plot selection included ease of access, with sites being chosen that were within 100 m and greater than 30 m of established trails. Sites were selected within 50 m from rivers and water bodies to minimize the influence of local topography and additional effort required when measuring forested plots in wetlands. There are also a great deal of permanent plots at La Selva, so to avoid disturbing ongoing long term research, we selected plots that they were at least 25 m away from established study areas. Locations of random plots did not require specific permission by the reserve, nor did our field research involve any endangered or protected species. Field plots were located using a Garmin 76CSx GPS.

#### Field biometric measurements

For each of the twenty plots we measured stand and tree attributes using a variable plot design. Trees were counted using a Spiegel-relaskop using a basal area factor (BAF) of 4 m^2^ ha^-1^ at the plot center and at four satellite plots spaced 30 m from the plot center on each of the cardinal directions [47]. We chose this sampling method because the variable plot radius design allows for a stratified sampling of trees that are more likely to contribute to the canopy above a specific point. For sampled trees, we measured diameter at breast height (dbh) using a diameter tape, total height, and height to the base of the live crown for all trees using a Vertex hypsometer (Haglof Inc.). Buttressed trees that proved difficult in the measurement of dbh at the usual height (1.37 m) were measured directly above the buttresses optically using the Spiegel-relaskop at a known distance from the tree [48]. This method has been found to be more efficient and just as accurate in estimating DBH [48].

We conducted our field measurements in January 2012. We sampled plots located in old growth, abandoned pasture (approximately 70 years), and logged and secondary forest types. Basal area (area of the cross section of trees at breast height per total area) was measured on all 20 plots and four additional plots for each of the twenty primary plots for a total of 80 satellite plots. These 80 satellite plots also used the same variable size plot design. Our stratified sampling design yielded trees of all dbh sizes classes, thus providing a good indication of canopy contribution for comparison with lidar data. The quadratic stand diameter (QSD) was also calculated from plot-level summary data. This is determined from the equation from [49]:

$$QSD=\sqrt{\left(\frac{BA}{N}\right)*\left(\frac{4}{\pi }\right)}$$

with BA–Basal area, and N–number of stems. Total estimated biomass for each tree was calculated using two different sets of allometric equations [50–51]. Average stand properties were calculated following adjustment of individual trees for their sampling frequency [49]. All estimates were calculated on a per-hectare basis and weighted according to our stratified sampling design, *i*.*e*., basal area [52]. Sampling trees with probability proportional to basal area allows for the mean height of the measured trees to provide an unbiased estimate of Lorey’s height, which is a plot-level basal area weighted mean height. Plot locations and measured biometric properties were presented in [19].

#### Terrestrial Laser Scanner (TLS)

**Fig 1:**
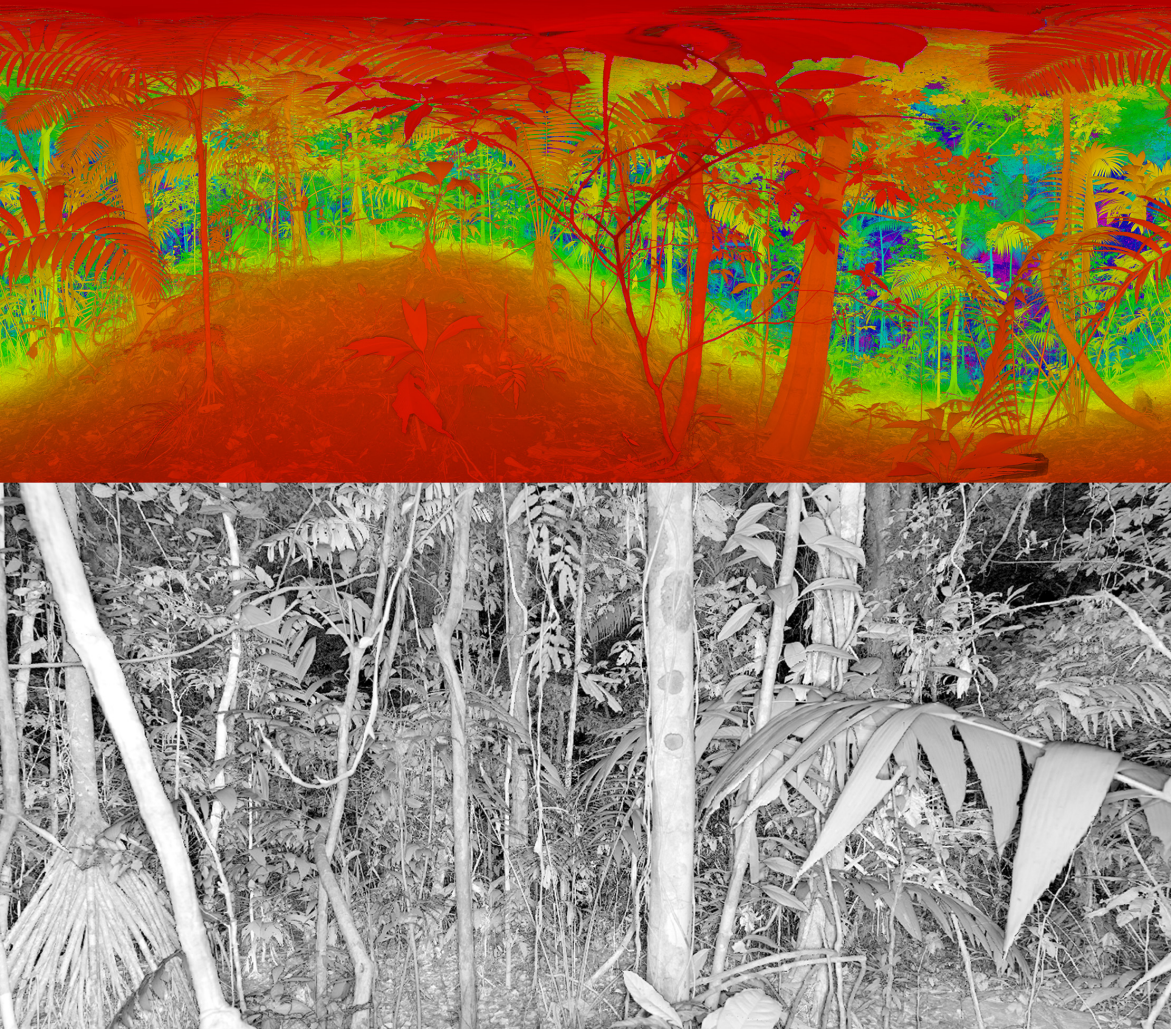
Terrestrial based lidar (TLS) scans of tropical forest on a hillslope at La Selva Biological Reserve, Costa Rica. Top-Color indicates distance from scanner, while saturation indicates laser reflectivity. Distortion of canopy elements near the top of the image is due to cylindrical reprojection of a hemispherical scan. Note that the image here is a 100x downsampling of the original scan, which includes over 40 million (*x*,*y*,*z*) coordinates. Bottom-Higher resolution TLS image focusing on the understory, white indicates closer objects, black indicates more distant returns.

We used a FARO Focus 3D for our TLS scans, with a narrow beam width (~5mm at 50m range). The TLS returns approximately 40 million points per plot. Scans were conducted in the center of our field measured plots. Examples of terrestrial lidar scans are presented in Fig 1. This instrument weighs 5.2 kg, and is deployed on a light-weight carbon fiber tripod. It is self-contained and transported in a weatherproof field case, and capable of operating on a single internal rechargeable battery to conduct multiple scans over a full field day. Data is collected on a Secure Digital card (SD), allowing for an entire day of field data to be stored.

Scans were recorded in the center of our field-plots. When single-scan TLS is employed to recover the size, characteristics, and position of individual tree stems, occlusion by other vegetation can present significant challenges, leading to non-detection bias ([53–54] for corrective techniques). However, in this study, the focus is on the recovery of bulk canopy attributes, and we employ a statistical approach based on a modified MacArthur and Horn estimator [55], that specifically accounts for occlusion, described in section 2.2.1 Relative vegetation profiles.

### Processing

#### Relative vegetation profiles

To construct vertical profiles of plant surface area from TLS scans, we adopted a quasi-likelihood approach, following [56]. The approach builds on the connection between the MacArthur-Horn estimator [55] and the family of statistical techniques known as survival analysis [57].

Consider a volume element defined in 3-dimensional space, penetrated by *n* TLS probes. Let β_i_ denote the angle of elevation of the i^th^ probe above the horizontal plane. We model the distribution of plant surfaces using a set of *m* discrete classes; denote the inclination of the *j*^th^ class relative to the horizontal as α_j_, and its density (m^2^/m^3^ of projected surface area) as ρ_j_. We assume either that the distribution of surface angles is radially symmetrical, or that the distribution of probes is; the latter assumption is appropriate for tripod-mounted TLS with volume elements centered over the tripod position. Then the apparent density of surfaces, normal to a probe with angle β_i_, is

$$F_{\beta_{i}}=\sum_{j}g_{\alpha_{j},\beta_{i}}\rho_{j}$$

where (following [58])

$$g_{\alpha_{j},\beta_{i}}\;=\;\text{cos}\;\alpha_{j}\;\text{sin}\;\beta_{i}\;\alpha_{j}\leq \beta_{i}$$

$$g_{\alpha_{j},\beta_{i}}=\frac{2}{\pi }\text{sin}\;\alpha_{j}\;\text{cos}\;\beta_{i}\;\text{sin}\;\theta_{0}+\left(1-\frac{\theta_{0}}{90}\right)\;\text{cos}\;\alpha_{j}\;\text{sin}\;\beta_{i}\;\alpha_{j}\leq \beta_{i}$$

and

$$\theta_{0}={\text{cos}}^{-1}\left(\text{cot}\;\alpha_{j}\;\text{tan}\;\beta_{i}\right)$$

Now, let l_i_ be the length of the i^th^ probe within the volume element (originating at the TLS unit, or on a boundary surface of the volume element; and terminating either on contact with a surface, or by exiting the volume element). Let d_i_ = 0 indicate that the i^th^ probe contacted a surface, and d_i_ = 1 indicate that it exited the volume element without contact. Then, treating the *n* probes as independent observations, following the general model of [56] the log-likelihood of the observed data can be written as

$$\text{ln}\;L=\sum_{i=1}^{n}-l_{i}F_{\beta_{i}}+(l-d_{i})\;\text{ln}\left(F_{\beta_{i}}\right)$$

We used cylindrical volume elements 1 m thick, and 20 m radius, centered on the tripod position and ranging from the ground surface to the maximum observed tree height. Within each volume element, the plant surface density was estimated by maximizing the likelihood equation, subject to the constraint that ρ_j_≥0 for all classes, using m = 9 angle classes (centered on 5, 15, 25, …, 85 degrees from the horizontal). The horizontal projection of the surfaces within each angle class was then summed to yield the vertical distribution of horizontally-projected plant surfaces.

#### Parameters from RVPs

From RVPs, we calculated a series of metrics for developing statistical relationships between lidar data and field-based vegetation structural components (Table 1), which we used previously in a study comparing canopy profiles developed from airborne lidar [19]. The metrics we used for our analysis include a profile layer count, peak maxima (i.e. height above ground of the largest maximum), highest maxima (i.e. height above ground of the highest maximum), profile median height, and a ratio of median height to maximum height of each profile. In addition, we calculated entropy [19, 59], and lidar coherence of Fourier transforms, using Fast Fourier Transforms (FFT) at frequencies of 0.087 rad m^-1^, 0.15 rad m^-1^, 0.31 rad m^-1^, 0.46 rad m^-1^, 0.67 rad m^-1^, and 1.04 rad m^-1^ [60], which have been shown to be correlated with biomass when using airborne lidar data. These frequencies correspond to so-called vertical wavelengths of 73, 42, 20, 14, 9, and 6m, respectively. FFT parameters do not include phase.

**Table 1:** Description of lidar-derived vertical profile metrics.

Variable	Description
Mean Synthetic DBH	mean dbh of modeled trees in synthetic forest
Synthetic Shape	shape parameter of the best fit Weibull distribution
Coh_0.087	lidar coherence at a frequency of 0.087 rad/m (73 m vertical wavelength)
Coh_0.15	lidar coherence at a frequency of 0.15 rad/m (42 m vertical wavelength)
Coh_0.31	lidar coherence at a frequency of 0.31rad/m (20 m vertical wavelength)
Coh_0.46	lidar coherence at a frequency of 0.46 rad/m (14 m vertical wavelength)
Coh_0.67	lidar coherence at a frequency of 0.67 rad/m (9 m vertical wavelength)
Coh_1.04	lidar coherence at a frequency of 1.04 rad/m (6 m vertical wavelength)
Entropy	forest height diversity within 1 m bins
PAI	estimated plant area index
Layer Count	number of local maximums in vertical profile
Highest Maxima	elevation of the highest local maximum
Layer Diff	elevation difference between highest maxima and lowest maxima

#### Theoretical stands, synthetic forests and vegetation profiles

Tree height, crown geometry, light dynamics, and canopy foliage have been linked to tree trunk diameters through allometric equations [59, 61–65]. Through the comparison of tree trunk diameter groups, specific insight may be gleaned in regard to growth and disturbance dynamics [1, 66]. Ratios comparing successive diameter classes tend to be consistent for a forest that is considered to be at or near a steady state [67–68]. These ratios are often termed q-ratios because of the "quotient of diminution" or rate of change between diameter classes [68–69]. A constant q-ratio is also expressed as an exponential diameter distribution [11, 67, 68, 70]. It was determined that q varied within stands [69], despite much literature focusing on a constant q in mixed age states or exponential distribution. The Weibull distribution includes the constant-q as a special case when the shape parameter is set to one [71].

Previously, we developed synthetic forest algorithm that uses geometric series to generate forest stands [19, 72]. Our model uses allometric equations that relate crown size to dbh [12, 73]. Using a random tree trunk size pulled from a Weibull distribution, we place the tree on the landscape (horizontal location). For each tree placed on the landscape, we generate an ellipsoid in three dimensional space based on these parameters (dbh and crown geometry) to develop a forest canopy. If there is too much overlap, i.e. crown shying or light competition, we determine another random horizontal location for the tree and repeat the check on crown overlap. Crown overlap of less than half of the horizontal radius of any crown was used in our model and results in field-based measurements of gap values [72, 74]. Parameters used in our synthetic forest model are found in Table 2. Our method for developing a vegetation profile based on theoretical stand information is still novel and is explored in [19, 21], and utilized for stand metrics in [72]. We note that other scientific disciplines have used theoretical models of physically based systems to interpret observed phenomena, such as exoplanet ring systems [75–76].

**Table 2:** Synthetic forest parameterization.

Parameter	Value or Equation
area of simulation	1 km^2^
mean of the distribution (α)	8–150 cm (binned in 1 cm intervals)
shape parameter of distribution (β)	0.8–1.2
range of stem diameter distribution	0–500 cm
spacing between tree crowns	1/2 crown of existing trees
number of trees in distribution	200,000
crown geometry allometric equations	Asner et al., 2002, Palace et al., 2003

**Fig 2:**
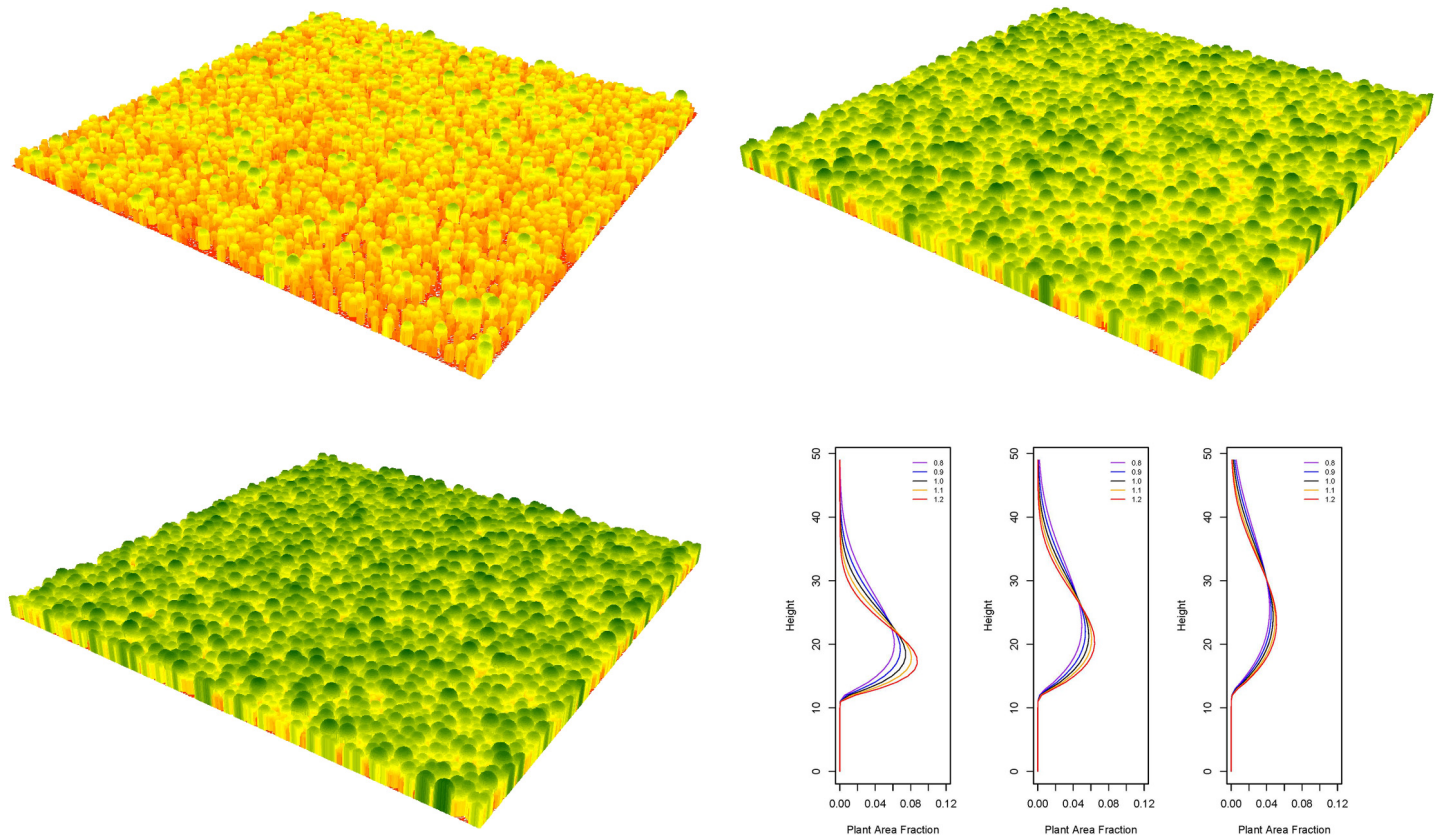
Examples of synthetic forests, with areas representing 1 km^2^. Colors indicate height at top of the canopy. Shown here are forests and profiles with mean diameters of 15 cm (left), 30 cm (middle) and 55 cm (right), with the shape parameter of the Weibull distribution varied from 0.8 to 1.2 in increments of 0.1.

Three-dimensional synthetic forest canopies were aggregated to represent vertical profiles derived from Weibull attributes (Fig 2). We generated thousands of synthetic forests and their resulting vertical profiles. Once normalized the relative vegetation profiles were compared with the lidar RVPs. A goodness-of-fit was used to determine which synthetic vegetation profiles best matched a lidar RVP. The Weibull distribution parameters represented by the best fit synthetic forest profile were then used in the development of multiple linear regressions along with other parameters (entropy, PAI, FFT, etc.). We stress that these parameters were representative of theoretical forests and provided an additional means to characterize terrestrial lidar information. Currently, our model is able to ingest spatial point data from field plots and develop a three dimensional canopy. In our efforts to develop theoretical forests stands, we rely on randomly pulling a tree diameter from a distribution and then randomly placing it across the landscape, using crown overlap as the spacing mechanism. There are other approaches to modeling efficient tree and crown spacing, such as spatial point processes, but we have opted for a simpler approach for this model.

### Statistics and Computational Coding

Our estimates of theoretical forests and the associated synthesized vegetation profile utilized code developed in Python 2.7 (Python Software Foundation, Python version 2.7, www.python.org). Multiple regression models using forward stepwise regression with Bayesian information criteria (BIC) for variable inclusion in the model were developed using field-measured forest structure and terrestrial lidar metrics [19, 77], including estimates of forest gappiness, canopy layers, and comparisons with our theoretical forest vegetation profile. We also used the FFT analysis for in our model development. Numerous other variables were calculated for this study, such as percentile scores for the RVP, however we specifically chose variables to explore that could be interpreted as biophysical attributes of the vegetation profile. Python 2.7 was used to derive lidar metrics and for a least-squares analysis to compare theoretical profiles with TLS derived profiles. We used JMP10 Pro (www.jmp.com) for regression model development. Multiple regression analysis is specifically designed to accommodate correlated variables; there is no requirement that the variables be orthogonal. We used Bayesian information criterion in our stepwise variable selection. This is a method to allow for variable inclusion, but not at the expense of overfitting, by including a penalty term for the number of parameters in the model. The use of stepwise should only select variables that add to building a more significant model and parameters that are highly correlated may only have one of the variables included due to the second correlated variable not contributing to the model’s performance.

## Results

### Field-based Measurements

Field-measured forest structural data for the twenty plots were presented in [19]. We note that the forests examined in [19] and in this paper are high biomass and tall statured ranging in biomass from 190.3 to 362.4 Mg ha^-1^ and average tree height from 10.12 to 39.20 m.

### Lidar Metrics

Metrics derived from the RVPs generated from the terrestrial lidar point cloud data are presented in Table 3. Metrics are discussed here in the range of estimates, as well as the mean and standard deviation. Metrics derived from synthetic profiles developed from our theoretical forest model for mean dbh ranged from 11.35 to 75.39 cm and the shape of the profile showed little different with only plot BP8, not being a 0.8, but rather 0.9. We stress that these values are not specifically comparable to field measured values, but provide a method to cull more information from the RVP generated from the ground-based terrestrial lidar data.

**Table 3:** Vertical profile derived lidar metrics.

plot	synth_actual	synth_shape	ft_73m_0.087	ft_42m_0.15	ft_14m_0.46	ft_9m_0.67	ft_20m_0.31	ft_6m_1.04	entropy	PAI	gapfrac	max_layer	layer_diff	layer_count	highmax
BP1	36.14	0.8	0.624	0.138	0.021	0.146	0.665	0.149	3.12	3.50	0.030	1	41	5	42
BP10	58.72	0.8	0.732	0.488	0.340	0.342	0.228	0.090	3.18	3.46	0.031	1	49	8	50
BP11	58.72	0.8	0.853	0.656	0.138	0.107	0.332	0.083	3.09	2.97	0.051	8	48	7	49
BP2	14.76	0.8	0.812	0.519	0.129	0.285	0.219	0.115	3.14	3.62	0.027	1	49	6	50
BP3	11.35	0.8	0.758	0.441	0.259	0.143	0.125	0.151	3.27	3.52	0.030	1	49	7	50
BP4	14.76	0.8	0.835	0.571	0.099	0.339	0.166	0.207	3.05	3.13	0.044	1	48	6	49
BP5	72.42	0.8	0.896	0.742	0.297	0.158	0.441	0.139	2.86	3.59	0.028	2	46	7	48
BP6	75.39	0.8	0.541	0.587	0.348	0.308	0.488	0.252	3.17	2.77	0.063	1	47	7	48
BP7	27.19	0.8	0.877	0.686	0.236	0.111	0.317	0.090	3.05	2.87	0.057	6	40	5	41
BP8	12.62	0.9	0.786	0.447	0.197	0.342	0.317	0.186	3.13	2.90	0.055	1	46	8	47
BP9	53.97	0.8	0.644	0.244	0.145	0.050	0.443	0.065	3.33	3.31	0.036	1	44	5	45
GL1	46.86	0.8	0.714	0.411	0.281	0.249	0.259	0.295	3.20	2.53	0.080	1	48	6	49
GL2	11.35	0.8	0.750	0.383	0.368	0.277	0.330	0.167	3.15	3.18	0.041	1	46	7	47
GL3	36.89	0.8	0.723	0.410	0.329	0.322	0.540	0.294	2.99	3.46	0.032	1	45	5	46
GL4	50.86	0.8	0.750	0.475	0.230	0.212	0.472	0.220	3.08	3.73	0.024	1	41	5	42
GL5	14.76	0.8	0.819	0.547	0.394	0.211	0.376	0.098	3.01	3.58	0.028	1	39	6	40
GL6	75.39	0.8	0.706	0.515	0.204	0.339	0.439	0.309	2.93	4.16	0.016	1	43	5	44
GL7	29.41	0.8	0.639	0.195	0.071	0.201	0.419	0.177	3.31	3.66	0.026	1	48	6	49
GL8	75.39	0.8	0.887	0.712	0.280	0.372	0.153	0.133	2.85	3.76	0.023	1	47	7	48
GL9	27.19	0.8	0.861	0.640	0.200	0.180	0.221	0.033	3.09	2.48	0.084	4	42	5	46
min	11.35	0.8	0.54	0.14	0.02	0.05	0.13	0.03	2.85	2.48	0.02	1	39	5	40
max	75.39	0.9	0.90	0.74	0.39	0.37	0.67	0.31	3.33	4.16	0.08	8	49	8	50
mean	40.21	0.8	0.76	0.49	0.23	0.23	0.35	0.16	3.10	3.31	0.04	1.80	45.30	6.15	46.50
st. dev.	23.66	0.02	0.10	0.17	0.10	0.10	0.14	0.08	0.13	0.44	0.02	1.94	3.25	1.04	3.17

Transformed RVPs were used for lidar metrics except the FFT analysis which used untransformed data. This was done to follow the methodology used in [60] and then in [19]. Fourier transforms for a coherence of 0.087 rad m^-1^ coherence (vertical wavelength of 73 m) ranged from 0.54 to 0.90. At a frequency of 0.15 rad m^-1^ coherence (vertical wavelength of 42 m) ranged from 0.14 to 0.74. Values ranged from 0.02 to 0.39 for a frequency of 0.46 rad m^-1^ coherence (vertical wavelength of 14 m). Fourier amplitudes for a coherence of 0.67 rad m^-1^ coherence (vertical wavelength of 9 m) ranged from 0.05 to 0.37. Values ranged from 0.13 to 0.67 for a frequency of 0.31 rad m^-1^ coherence (vertical wavelength of 20 m). Amplitudes from the Fourier transforms for a coherence of 1.04 rad m^-1^ (vertical wavelength of 6 m) ranged from 0.03 to 0.31.

Entropy estimates from the lidar derived and transformed RVP ranged from 2.85 to 3.33, with the higher value indicating both a canopy that has more depth and that is more complex in layering. PAI ranged from 2.48 to 4.16 with the upper bound indicating more plant material over a given plot in the trees and canopy, but with no differentiation between leaves, stems, or branches. Gap fraction ranged from 0.02 to 0.08 with the higher value indicating a greater change of canopy penetration of light and the possibility of either disturbances or multi-layered nature of the forest. The number of estimated canopy layers ranged from 1 to 8, with the higher value representing a more complex canopy with a number of different tree crowns at differing heights. The height difference in the layers in meters ranged from 39 m to 49 m, with this metric being an indication of canopy depth. If only one layer was estimated this was the depth of that layer, and if multiple layers were found, this was the depth of all layers combined. Finally, we found that the height of the highest layer ranged from 40 to 50 m, indicating a rather tall statured forest.

### Multiple Linear Regressions

**Fig 3:**
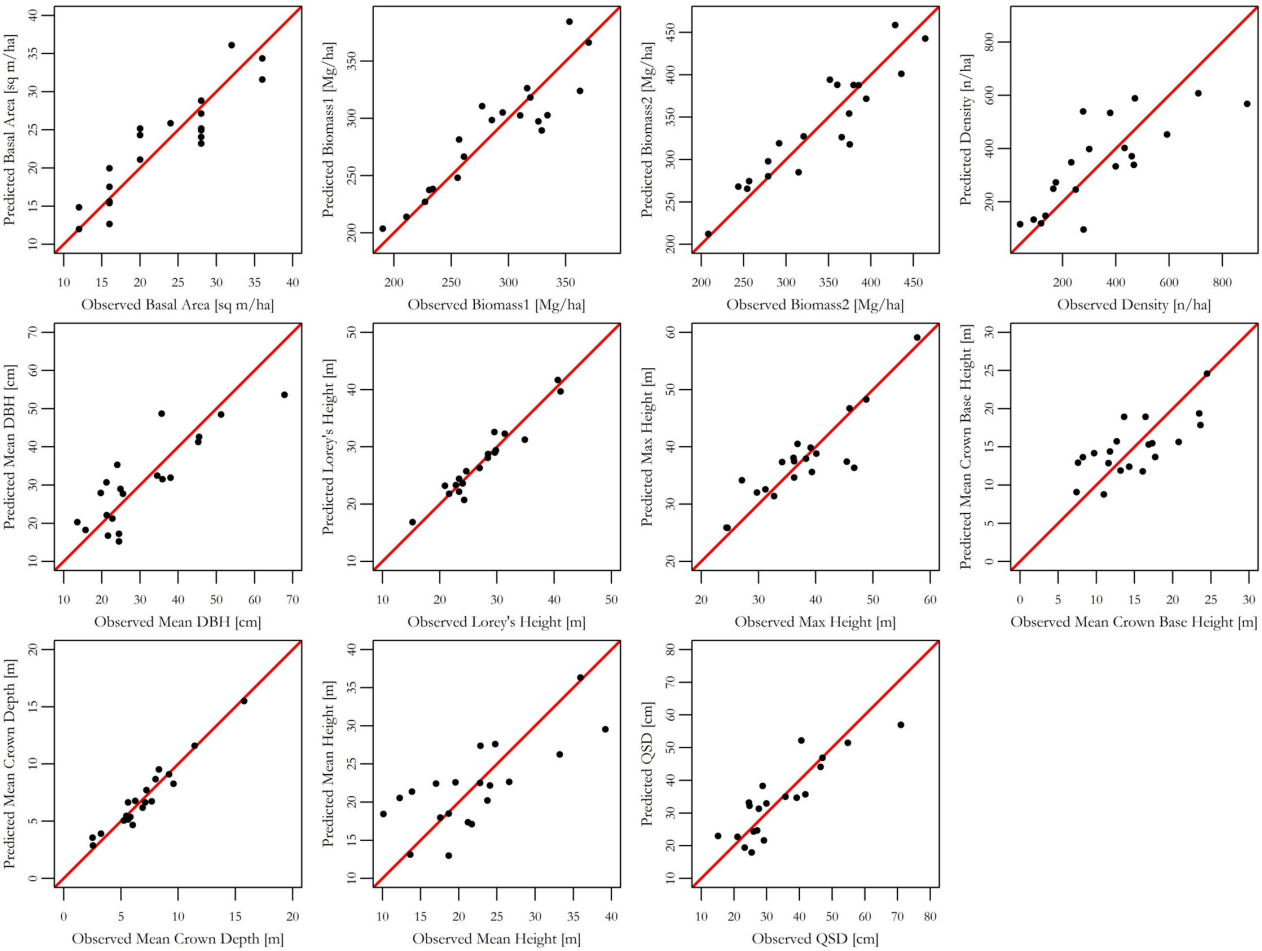
Observed verses predicted forest biometric properties based on multiple linear regression models using stepwise variable selection.

We developed a multiple linear regression using forward stepwise variable selection with BIC criteria for variable inclusion (Fig 3). A regression was developed for each of the field-measured or derived forest structural properties (Table 2). Model results are presented in Table 4 and present estimators of lidar metrics, adjusted r-squared values, significance of the model, and root mean squared error (RMSE). Moderate to strong relationships between field-measured traits and a suite of metrics derived from the vegetation profiles we developed using lidar data (lidar metrics) were found with all models converging on a statistically significant model to estimate specific forest structural attributes.

**Table 4:** Forest biometric properties and estimators from lidar metrics.

	Basal Area	Biomass1	Biomass2	Density	Lorey's height	Max height	Mean Crown Base Height	Mean Crown Depth	Mean DBH	Mean height	QSD
**Intercept**	*97*.*14*	*767*.*07*	**1555.18**	*2985*.*36*	***-201*.*95***	***65*.*42***	***53*.*78***	***-151*.*13***	***35*.*91***	***56*.*14***	***37*.*87***
**synth_actual**	**-**	*1*.*09*	*1*.*27*	**-6.26**	*0*.*08*	**0.14**	**-**	**-**	**0.26**	**-**	**0.28**
**synth_shape**	*-108*.*21*	-612.95	**-**	-3264.19	**-**	**-**	**-**	**-42.26**	**-**	**-**	**-**
**ft_73m_0.087**	30.04	*407*.*18*	**-**	**-**	**-**	***-53*.*38***	**-35.11**	**19.68**	**-**	***-52*.*14***	**-**
**ft_42m_0.15**	*-31*.*62*	***-393*.*79***	**-672.96**	**775.82**	**-**	**-**	**-**	**-**	***-65*.*37***	**-**	***-64*.*60***
**ft_14m_0.46**	**21.56**	*201*.*34*	**414.57**	**-**	*14*.*89*	20.88	**-**	**-**	**-**	**-**	**-**
**ft_9m_0.67**	*24*.*08*	**-**	**-**	**-**	**-**	-18.72	**-**	**-**	**-**	**-**	**-**
**ft_20m_0.31**	**28.89**	*134*.*98*	**-**	**-**	**28.03**	**-**	**-**	***30*.*33***	**-**	**-**	**-**
**ft_6m_1.04**	***-57*.*29***	**-345.63**	**-612.87**	**-**	**-**	**-**	**-**	***-39*.*55***	**-**	**-**	**-**
**entropy**	**-**	**-**	*-366*.*77*	**-**	**22.78**	**-**	**-**	***-***	**-**	**-**	**-**
**PAI**	**-**	*-46*.*32*	-48.12	**-**	**27.96**	**-**	-3.68	***31*.*33***	**-**	**-**	**-**
**gapfrac**	**-**	**-**	**-**	-3564.75	**784.78**	*165*.*24*	**-**	***94*.*51***	***409*.*54***	133.68	***407*.*50***
**max_layer**	**-**	**-**	*17*.*16*	**-**	**-**	**-**	**-**	***-1*.*33***	**-**	**-**	**-**
**layer_diff**	**-**	**-**	**-**	**-**	**3.35**	**-**	**-**	***6*.*07***	**-**	**-**	**-**
**layer_count**	**-**	**-**	-20.76	**-**	**-**	**-**	**-**	***-***	**-**	**-**	**-**
**highmax**	**-**	**-**	**9.90**	**-**	*-2*.*87*	**-**	**-**	***-518***	**-**	**-**	**-**
**r**^**2**^	0.75	0.72	0.72	0.50	0.88	0.71	0.45	0.88	0.66	0.49	0.70
**p-value**	<0.001	<0.01	<0.01	<0.01	<0.001	<0.001	<0.01	<0.001	<0.001	<0.01	<0.001
**RMSE**	3.76	27.56	37.51	153.28	2.18	4.51	3.91	1.04	7.85	5.45	7.29

*p<0*.*001*

p<0.01

*p<0*.*05*

p>0.05

r^2^ values presented are adjusted r^2^ values because of inclusion of additional variables.

We found a significant relationship between basal area and lidar metrics (*r*^*2*^ = 0.75, *p* < 0.001, *RMSE* = 3.76 m^2^ ha^-1^). There was a significant, strong relationship between metrics and both of the biomass estimates with both models having the same r-squared value and lower RMSE for biomass 1 (biomass1: *r*^*2*^ = 0.72, *p* < 0.01, *RMSE* = 27.56 Mg ha^-1^; biomass2: *r*^*2*^ = 0.72, *p* < 0.01, *RMSE* = 37.51 Mg ha^-1^). We found a relationship between three parameters (FFT coherence (0.087), synthetic shape, and synthetic dbh) and tree density (*r*^*2*^ = 0.50, *p* < 0.01, *RMSE* = 153.28 trees ha^-1^). We also found that models developed to estimate Lorey’s height and maximum height were both significantly related to several lidar metrics. Lorey’s height and maximum height shared four variables in the final model, with each model having an additional metric included (Lorey’s height: *r*^*2*^ = 0.88, *p* < 0.001, *RMSE* = 2.18 m; max height: *r*^*2*^ = 0.71, *p* < 0.001, *RMSE* = 4.51 m). Mean crown base height was our least strong model, but still significant. Relationships were found between the mean crown base height and two lidar metrics (FFT coherence (0.087) and PAI (*r*^*2*^ = 0.45, *p* < 0.01 *RMSE* = 3.91 m). The strongest significant relationship was found for mean crown depth (*r*^*2*^ = 0.88, *p* < 0.001, *RMSE* = 1.04 m). Lidar metrics for quadratic stand diameter and mean dbh were found to develop significant relationships (*r*^*2*^ = 0.70, *p* < 0.001, *RMSE* = 7.29 cm; *r*^*2*^ = 0.49, *p* < 0.01, *RMSE* = 5.45 m^2^).

## Discussion

In this study, we collapsed the three-dimensional point cloud from a TLS into a two-dimensional canopy profile. We were able to predict many forest structural attributes, thus confirming the usefulness of a TLS for forest inventory (Fig 3). Our work indicates that a terrestrial lidar sensor can provide useful information on forest structure [19] and comparison can be readily made between different sensors, in this case TLS, and field-measurements as shown in [21]. Still, the reduction to a two-dimensional data source loses a great deal of spatial information that might be useful for discerning additional forest structure, particularly in the understory. TLS data can be reanalyzed with new algorithms and statistical analysis and can prove useful to examine new avenues of scientific questioning. This provides a unique opportunity to provide a snapshot of a forest inventory plot, allowing for reexamination, analysis, and check on recorded information.

The three dimensional canopy can be integrated and expressed in two dimensions using a vegetation profile, which is a model of the distribution of vegetation as a function of height. We found that metrics derived from these profiles provide insight into biometric properties, ranging from biomass to density of trees. We were able to develop significant relationships between these profile metrics and forest structural attributes. There were numerous variables that could be used for the development of multiple regression models, but for this analysis we chose to examine information derived from our theoretical stand model and lidar profile parameters not typically used in forest biometric studies.

The variable radius plot design with a stratified sampling approach that we used in this study proved advantageous both for developing relationships between TLS data and biometric properties, and for conducting field measurements. We stress that the variable radius plot design provides a rapid and rigorous assessment of the forest structure. This is because it does not over sample smaller trees, allows for the inclusion of trees that contribute to canopy structure, and provides estimates of basal area and its related properties, such as Lorey’s height, which are often used in remote sensing efforts. This approach made field work efficient and effective, evidenced by rapid field data collection (approx. 1 hour per plot). The TLS took fifteen minutes to collect, with new instruments providing high resolution and faster collection times. We suggest that the TLS is a highly useful and robust technique for quantifying forest structure, specifically in a tropical forest, where field effort is often high. We also note the usefulness of a TLS in conjunction with field plot measurements.

Field-based measurements were presented in [19] and are on par with those found from other studies at La Selva [60, 78–80]. In addition, our measured canopy height was consistent with that found in [28]. We found that many of the geometric canopy properties have not been measured in the field at La Selva, but were comparable to results from tropical forests in Amazonia and the pan-tropical regions [12, 63, 64, 73]. We stress that measurements other than just height are important in the understanding of forest dynamics and should be included in inventory efforts [19, 78, 80]. We note that the forests examined in [19] and in this paper are higher biomass tropical forests with less range in biomass than some other studies. The limited range in both height and biomass complicated our efforts, but nevertheless we were able to develop significant statistical models. We suggest that our approach could be used to improve and refine biomass estimation for REDD+ efforts.

### Theoretical forest stands and synthetic vegetation profile

The synthetic vegetation profile allowed for theoretical stand distributions to be compared with vegetation profiles developed from discrete return lidar data. This was specifically used to retrieve additional information about stand size class distributions from the lidar profile. In essence, the synthetic forest algorithm develops numerous vegetation profiles based on two parameters from a theoretical model. The two parameters (shape and mean of a forest dbh distribution) drive the modeled stand conditions, and therefore the thickness of the vegetation profile and the overall height. This is because dbh and height of vegetation are related, but other forest structural properties are more complex than just maximum height. The distribution of trees in various dbh classes represented by the Weibull distribution used in our synthetic forest model represent the canopy structure and vegetation profile of hypothetical forests.

Many different parameters derived from lidar vegetation profiles provided information toward estimating the mean canopy depth of a stand. The shape of the diameter distribution of the synthetic forest (synth_shape) provided information on the tree trunk size ratios between binned classes. In this study, we represented stand diameter distribution as a Weibull distribution. The shape parameter, therefore, offered an indication of the proportion of large to small trees. A lower number value for the shape of the synthetic profile indicates a forest with a greater proportion of larger to smaller trees, though the smaller trees may still outnumber the larger trees. This indicates a more complex forest stand structure consisting of a mix of larger and smaller trees, similar to primary forest with high stand density, which would have a complex and tall canopy. The resulting shape of the vegetation profile from lower synth-shape values is a narrower vertical distribution within canopy, with the profile maximum occurring at higher or lower canopy heights based on the mean dbh (synth_actual). We note that the shape of the best-fit synthetic profile was only different for one plot (BP8), and this variable may have been included in models because it captured the influence of that one plot in the regression model. The BP8 plot was located in an abandoned plantation. This plantation was abandoned over 70 years ago, and the biomass of the plot is on par with other old-growth plots in our study, as well as other forest parameters. Still, a combination of these forest attributes may indicate a different distribution of tree sizes reflected in the synth_shape in our theoretical forest. It is encouraging that our regression can adequately estimate stand density and other forest structural properties in forests that have large biomass values and forest metrics similar to old-growth forests. The alteration of the proportion of tree stand size classes may indicate a disturbance [66]. Other regressions that utilized the shape parameter for the synthetic profile are the basal area, biomass2, and mean crown depth. The results of this study with regard to theoretical forest stand profiles warrant further investigation of the use of this approach for interpretation of forest disturbance and past human activity. We suggest the use of this model comparison approach to aid in the possible interpretation of forest disturbance and past human activity.

### Fast Fourier Transforms and other Canopy Profile Metrics

Magnitudes of coherence of Fast Fourier Transforms of canopy height profiles provide insight into the canopy structure [19, 81]. The frequencies used in this study correspond to wavelengths that are structurally significant in La Selva and have been shown to be particularly useful for estimating biomass using airborne lidar and radar [60]. For example, mean canopy depth at La Selva averages around 7 m and mean canopy height averages around 22 m [19]. The coherence of the frequencies corresponding to these wavelengths were significant in our analysis, as has been shown in previous studies [19, 60, 81]. This can provide some indication of the structurally significant features that are present in the canopy height profile. In terms of biomass estimation, characterizing the structure of the canopy by coherence of frequencies corresponding to features like depth and canopy height can serve as an analog for the amount of canopy material present. In previous studies, lidar coherence was calculated using canopy profiles derived from airborne lidar data. It is notable that in this study we achieved similar results using terrestrial-based lidar systems, which implies that structural features present in canopy profiles can be observed using above or below canopy sensors. Our analyses included frequencies corresponding to vertical wavelengths of 73, 42, 20, 14, 9, and 6m, respectively. These echo strong forest height signals of approximately mean crown depth and mean tree height in our study.

### Merits of Multiple Linear Regressions

We found that the TLS provides good estimates of canopy properties when modeled using multiple linear regression of metrics derived from canopy profiles. The best performing model developed in this study was for mean crown depth (*r*^*2*^ = 0.88, *p* < 0.001, *RMSE* = 1.04 m). This model also includes the most variables in the construction of all of our multiple regressions. Although regression models containing many parameters are thought to be problematic due to overfitting, we stress that with BIC variable selection, this was not the case. BIC variable selection favors the exclusion of unnecessary variables from models. Model performance provided insight into the complexity of forest structure as measured using terrestrial lidar scans, and also hinted at how canopies are organized in forested ecosystems.

The regression model developed to estimate stand density (number per hectare) only include two lidar metrics, shape of the synthetic profile diameter distribution (synth_shape) and FFT coherence (0.087). The model also had a significant, but rather low r^2^ value compared to other models (*r*^*2*^ = 0.50, *p* < 0.01, *RMSE* = 153.28 n ha^-1^). The coherence at a frequency of 0.087 rad/m, corresponding to a vertical wavelength of 73 meters, was positively related to stand density. A taller forest in a primary forest often has more stems because the complex gallery forest allows for smaller trees to begin growing underneath in a multi-aged and tiered forest and canopy when compared to an even-aged forest of similar average height.

Our regression model to estimate biomass (Mg ha^-1^) performed very well (biomass1: *r*^*2*^ = 0.72, *p* < 0.01, *RMSE* = 27.56 Mg ha^-1^; biomass2: *r*^*2*^ = 0.72, *p* < 0.01, *RMSE* = 37.51 Mg ha^-1^). Fast Fourier Transform variables all were included except for coherence (0.31) for Biomass2 and the additional exclusion of coherence (0.087) for Biomass1. The inclusion of these variables indicates the importance of looking at FFTs in the estimation of biomass in tropical forests using TLS. These variables represent the relative layering and complexity of the forest canopy, with a mixed age stand exhibiting a more complex canopy and containing higher biomass due to the optimal space packing of canopy architecture. Improvements in the disordered packaging using ellipsoids have been explored and may be comparable to the crown geometric space filling of tree canopies [82–83]. Supporting this is also the inclusion of entropy of the RVP for the biomass1 model, which is indicative of the complexity of canopy vegetation distribution. PAI and maximum layer height were included for both biomass estimates. Biomass1 also included a negative relation with layer count and a positive relation with maximum height. The estimated dbh from the synthetically derived RVP was used in both regressions and biomass2 also included a negative relation with the shape of the synthetic RVP.

Biomass is often the structural characteristic of forests that is of highest interest due to REDD+ efforts, but this focus neglects additional forest structural information that can be used to infer the past and future trajectory of forest stands. The estimation of such characteristics, such as basal area, areal density, crown geometry, and size class distribution, are within reach using either airborne or terrestrial lidar systems. We focus on biomass because it is easier to estimate across a broad range of values, ranging from lower secondary estimates to higher full stature forests. However, much of the focus of biomass estimation efforts has been in higher biomass tropical forests, where remote sensing efforts can fall short due to instrument limitations (*e*.*g*. saturation) or issues with linking plot level estimates with moderate scale remote sensing image data. The estimation of biomass even within and across high biomass forests is possible with lidar data, but requires quality field-data for statistical model development. Further, lidar data are particularly amenable to biomass estimation in high biomass forests because of the wealth of information about forest stands that can be retrieved from lidar-derived vegetation profiles, which we presented in this paper.

## Conclusions

Our study examined the use of a TLS to estimate forest structure using vegetation profiles derived from the three-dimensional point cloud. In this paper, we described in detail the methodology for their calculation from TLS data. We developed a multitude of parameters from the vegetation profiles that were used in the development of multiple linear regressions. These parameters included entropy, PAI, number of layers, and those derived from FFT frequency analysis. In addition, we used information from theoretical forests exhibiting varying stand properties, primarily dbh size class distribution. This novel approach compared theoretical stand profiles to lidar vegetation profiles using a least-squares fit to infer stand characteristics using TLS data. We developed significant models for all the forest structure parameters. Different models included a variety of parameters derived from vegetation profiles. The FFT analysis proved the most utilized of all parameters in the different models of forest structure. The many attributes derived from vegetation profiles in our study and their inclusion in regression models to predict forest biometric properties is indicative of the complexity of tropical forest canopies. We suggest that it is important to utilize these derived parameters from the vegetation profiles and not just singular one such as total height, mean height or entropy. Though many of these forest structural attributes may be biome and species specific, generalizations made across larger tropical regions will allow us first to refine cruder biomass estimates, incorporate additional field-based measurements for comparison, and allow for parsing of simplified biome based estimates of forest structure, allowing for new avenues of research relating remotely sensing image data to plot specific understanding of biometric properties.
